# Supplementary material for: Microplastic-Enhanced Cadmium Toxicity: A Growing Threat to the Sea Grape, Caulerpa lentillifera
Source: Antioxidants (Basel). 2024 Oct 18;13(10):1268. doi: 10.3390/antiox13101268 (PMC11505291; doi:10.3390/antiox13101268)
Supplement: Supplementary file 1 [file antioxidants-13-01268-s001.zip › antioxidants-3249139-supplementary.pdf]

Supplementary Table S1

Formula of artificial seawater.

Component	Chemical Formula	Concentration (g/L)	Purity	Manufacturer
Sodium chloride	$NaCl$	24.53	Analytical grade	Shanghai Macklin Biochemical Technology Co., Ltd.
Magnesium chloride (hexahydrate)	$MgCl_2 \cdot 6H_2O$	11.1	Analytical grade	Shanghai Aladdin Biochemical Technology Co., Ltd.
Magnesium sulfate (heptahydrate)	$MgSO_4 \cdot 7H_2O$	3.3	Analytical grade	Shanghai Aladdin Biochemical Technology Co., Ltd.
Calcium carbonate	$CaCO_3$	1.6	Analytical grade	Guangzhou XiLong Scientific Co., Ltd.
Sodium bicarbonate	$NaHCO_3$	0.2	Analytical grade	Guangzhou XiLong Scientific Co., Ltd.
Potassium chloride	KCl	0.7	Analytical grade	Shanghai Macklin Biochemical Technology Co., Ltd.
Sodium bromide	$NaBr$	0.1	Analytical grade	Shanghai Aladdin Biochemical Technology Co., Ltd.
Sodium fluoride	NaF	0.003	Analytical grade	Shanghai Aladdin Biochemical Technology Co., Ltd.
Boric acid	H_3BO_3	0.026	Analytical grade	Guangzhou XiLong Scientific Co., Ltd.
Barium sulfate	$SrSO_4$	0.004	Analytical grade	Guangzhou XiLong Scientific Co., Ltd.

Supplementary Table S2

Data filtering and base information statistics.

Sample	Raw reads	Raw bases	Clean reads	Clean bases	Mapped reads	Mapped ratio (%)	Error rate (%)	Q20 (%)	Q30 (%)	GC (%)
**Control-1**	43095444	6507412044	42874592	6444824435	34888916	81.37	0.0117	98.93	96.44	42.34
**Control-2**	45307544	6841439144	45047794	6727149426	35461860	78.72	0.0116	99.00	96.69	40.32
**Control-3**	43581148	6580753348	43342528	6515313446	35865556	82.75	0.0118	98.88	96.32	42.16
**MPs-1**	46163718	6970721418	45919486	6858112857	36270714	78.99	0.0116	98.99	96.64	40.30
**MPs-2**	45111890	6811895390	44872870	6744508196	37016676	82.49	0.0118	98.92	96.43	42.09
**MPs-3**	42534056	6422642456	42337144	6359487944	34633326	81.80	0.0117	98.97	96.54	42.11
**Cd-1**	45050900	6802685900	44817716	6726897046	36298920	80.99	0.0117	98.94	96.50	40.81
**Cd-2**	49047238	7406132938	48789588	7327161516	40351650	82.71	0.0118	98.92	96.44	41.80
**Cd-3**	47617654	7190265754	47334556	7074966135	36379584	76.86	0.0117	98.97	96.59	40.24
**MPs+Cd-1**	42687136	6445757536	42439458	6342170986	34135380	80.43	0.0117	98.97	96.59	40.45
**MPs+Cd-2**	45321890	6843605390	45088990	6756198278	36249094	80.39	0.0117	98.98	96.62	40.63
**MPs+Cd-3**	49814314	7521961414	49492000	7400029605	39943688	80.71	0.0117	98.93	96.47	40.96
